# A Prediction Model Intended for Exploratory Laparoscopy Risk Stratification in Colorectal Cancer Patients With Potential Occult Peritoneal Metastasis

**DOI:** 10.3389/fonc.2022.943951

**Published:** 2022-07-13

**Authors:** Yuanxin Zhang, Xiusen Qin, Yang Li, Xi Zhang, Rui Luo, Zhijie Wu, Victoria Li, Shuai Han, Hui Wang, Huaiming Wang

**Affiliations:** ^1^ Department of Colorectal Surgery, The Sixth Affiliated Hospital, Sun Yat-sen University, Guangzhou, China; ^2^ Guangdong Provincial Key Laboratory of Colorectal and Pelvic Floor Disease, The Sixth Affiliated Hospital, Sun Yat-sen University, Guangzhou, China; ^3^ General Surgery Center, Department of Gastrointestinal Surgery, Zhujiang Hospital, Southern Medical University, Guangzhou, China; ^4^ Department of Secondary Education, Yew Chung International School, Kowloon Tong, Hong Kong, China

**Keywords:** exploratory laparoscopy, occult peritoneal metastasis, nomogram, colorectal cancer, decision curve analysis

## Abstract

**Background:**

The early diagnosis of occult peritoneal metastasis (PM) remains a challenge due to the low sensitivity on computed tomography (CT) images. Exploratory laparoscopy is the gold standard to confirm PM but should only be proposed in selected patients due to its invasiveness, high cost, and port-site metastasis risk. In this study, we aimed to develop an individualized prediction model to identify occult PM status and determine optimal candidates for exploratory laparoscopy.

**Method:**

A total of 622 colorectal cancer (CRC) patients from 2 centers were divided into training and external validation cohorts. All patients’ PM status was first detected as negative on CT imaging but later confirmed by exploratory laparoscopy. Multivariate analysis was used to identify independent predictors, which were used to build a prediction model for identifying occult PM in CRC. The concordance index (C-index), calibration plot and decision curve analysis were used to evaluate its predictive accuracy and clinical utility.

**Results:**

The C-indices of the model in the development and validation groups were 0.850 (95% CI 0.815-0.885) and 0.794 (95% CI, 0.690-0.899), respectively. The calibration curve showed consistency between the observed and predicted probabilities. The decision curve analysis indicated that the prediction model has a great clinical value between thresholds of 0.10 and 0.72. At a risk threshold of 30%, a total of 40% of exploratory laparoscopies could have been prevented, while still identifying 76.7% of clinically occult PM cases. A dynamic online platform was also developed to facilitate the usage of the proposed model.

**Conclusions:**

Our individualized risk model could reduce the number of unnecessary exploratory laparoscopies while maintaining a high rate of diagnosis of clinically occult PM. These results warrant further validation in prospective studies.

**Clinical Trial Registration:**

https://www.isrctn.com, identifier ISRCTN76852032

## Introduction

Despite recent improvements in cancer research, colorectal cancer (CRC) ranks second in mortality in both men and women worldwide (1). An important reason for the limited survival in CRC patients is the presence of distant metastasis. In particular, peritoneal metastases (PM) are associated with significantly shorter survival than metastases at other sites (p < 0.001) (2–4). The prediction of early PM plays an important role in the prognosis of CRC patients, because less aggressive cytoreductive surgery (CRS) is required for lower peritoneal cancer index (PCI) values and surgeons are also more likely to achieve complete (CC0) or near complete cytoreduction (CC1) (5). Both PCI and completeness of cytoreduction (CCR) are the indicators with the highest prognostic significance in the treatment of peritoneal carcinomatosis (6). All efforts should therefore be made to identify CRC patients with PM at the earliest stage.

Unfortunately, the early detection of colorectal PM is currently difficult due to the absence of typical symptoms and the low accuracy of noninvasive imaging methods for nodules smaller than 5 mm (7–9). Computed tomography (CT) is the most common noninvasive method to diagnose PM. CT detection of PM can only be suggested by omental caking, luminal narrowing, large nodules, and ascites (10). However, most of these PM-specific features usually exist at the late stage. Thus, CT detection of PM has high specificity but low sensitivity. This raises the problem that 10%-35% of CRCs with PM-negative status in CT readings were unexpectedly identified as PM-positive during subsequent surgery; also known as occult PM (11).

At present, diagnostic laparoscopy has been regarded as the most reliable tool to detect occult PM because it could provide direct visualization as well as histological confirmation, and could evaluate the extent of the disease, measured in terms of PCI (12–14). Meanwhile, some recent studies support the laparoscopy’s safety and efficacy either in the excision of lesions or in the selection of potential PM candidates for CRS/HIPEC (15–19). However, the laparoscopic approach should only be proposed to the selected patients due to its invasive procedure, high cost and the possibility of port-site metastasis. Therefore, it is essential to develop an individualized prediction model to identify occult PM status and determine optimal candidates for exploratory laparoscopy.

We aimed to develop a prediction model for the noninvasive prediction of occult PM status in CRC patients and study of its utility for exploratory laparoscopy risk stratification.

## Materials and Methods

### Patients

The clinicopathological characteristics of CRC patients with or without occult PM were retrospectively selected from a 2-center cancer dataset between September 2007 and July 2019. All enrolled patients were initially diagnosed as PM-negative on CT imaging but later confirmed to have the actual PM status during laparoscopy. The patients were divided into the following two cohorts: a training cohort (n = 552 from center 1) and an external validation cohort (n = 70 from center 2). The study was registered at ISRCTN (No. ISRCTN76852032). The study received approval from the local Institutional Review Committee (No. 2020ZSLYEC–109). The requirement to obtain consent from patients was waived due to the retrospective nature of the study. The work has been reported in line with the STROCSS criteria (20).

### Definitions and Variables

PM was defined as the dissemination of cancer cells in the abdominal or pelvic cavity, such as the greater omentum, ovaries, pelvic inlet, rectovesical (male) or rectouterine (female) pouch, and abdominal wall, or extensive carcinomatosis (21). These metastatic sites were determined to be malignant by pathological reviews of the biopsied or surgically resected specimens. All enrolled patients underwent enhanced CT examinations within two weeks before the operation. All CT images of patients were assessed and given a radiological diagnosis by at least two radiologists. A standardized exploration of the peritoneal cavity was conducted quadrant by quadrant using the endoscope, exploring the 13 regions of PCI as described by Sugarbaker (22). The procedures were only exploratory, and no extensive dissection was made (13, 23).

The baseline information of patients was extracted from the electronic dataset, including sex, age at diagnosis, primary tumor site, histological type, grade of differentiation, T stage, N stage, obstruction status, perforation status, carcinoembryonic antigen (CEA), carbohydrate antigen 125 (CA125) and carbohydrate antigen 19-9 (CA19-9) pretreatment levels, NRAS, KRAS, BRAF and PIK3CA gene mutation statuses, mismatch repair-deficient (dMMR) status, as well as systemic inflammatory markers such as the neutrophil-to-lymphocyte ratio (NLR), platelet-to-lymphocyte ratio (PLR) and lymphocyte-to-monocyte ratio (LMR).

### Inclusion and Exclusion Criteria

The inclusion criteria were as follows: (1) patients were diagnosed with CRC by endoscopy-biopsy pathology, combined with CT and/or other examinations; (2) patients had only one malignant primary tumor; (3) patients did not undergo the previous resection of the primary tumor; and (4) patients underwent both enhanced CT and exploratory laparoscopy.

The exclusion criteria were as follows: (1) typical PM signs on CT; (2) other distant metastases; and (3) previous inflammatory diseases.

### Construction of the Prediction Model

Univariate analysis was applied to assess the association between all included variables and occult PM. Statistical differences in the enrolled variables by PM status were assessed using the independent T test or Mann–Whitney U test for continuous variables and the Chi-square test or Fisher’s exact test for categorical variables.

Multivariate logistic regression analysis was adopted to identify independent risk factors for PM. According to the results of the multivariate analysis, a predictive nomogram was established to predict the risk of occult PM in CRC patients.

### Evaluation of the Accuracy and Utility of the Model

The receiver operating characteristic (ROC) curve (24) and calibration curve (25) were both used to evaluate the predictive accuracy of the model. On the one hand, the value of the area under the ROC curve (AUC) is the same as that of the concordance index (C-index) in a logistic regression model. The maximum value of the AUC is 1.0, indicating perfect discrimination, whereas 0.5 indicates a random chance to correctly discriminate the outcome with the model. On the other hand, the calibration curve graphically shows the relationship between the predicted and actual risks for each outcome. A plot that perfectly fits the 45° reference line would indicate good agreement.

Decision curve analysis (DCA) is a method for appraising prediction models and visualizing the clinical consequences of a decision strategy. DCA was carried out to evaluate the prediction model’s clinical application value by quantifying the net benefit and net reduction of each treatment strategy at different threshold probabilities (26, 27).

### Statistical Analysis

All statistical analyses were conducted with SPSS (version 25.0; IBM Corp., Armonk, New York, USA) and R software (version 4.1.2; http://www.r-project.org). Univariate analysis was performed with SPSS. The following R packages were used to perform multivariate logistic regression analysis and build the ROC curve, nomogram, calibration plot, DCA curve and dynamic online platform: “rms”, “pROC”, “rmda”, “dcurves”, “rsconnect” and “DynNom”. A two-sided P value <0.05 was used to indicate statistical significance.

## Results

### Patient Characteristics

A total of 662 eligible CRC patients from the 2-center cancer dataset were enrolled in the analysis. The development and validation cohorts included 552 and 70 patients, respectively. The demographics and tumor characteristics of the cohorts were comparable, as shown in **Tables 1**, **2**. In clinical practice, not all CRC patients have information on the status of NRAS, KRAS, BRAF, PIK3CA and dMMR. According to the maximum Youden index, the corresponding optimal cutoff values for NLR, PLR and LMR were 2.5, 172.1 and 2.6, respectively. Patient characteristics, including age, tumor location, histological type, grade of differentiation, T stage, N stage, obstruction status, perforation status, serum CA125 level, NLR, PLR and LMR, were significantly associated with occult PM after univariate analysis in the development cohort (P < 0.05).

**Table 1:** Characteristics of patients in the training and validation cohorts.

Characteristics	Training cohort			External-validation cohort		
	PM (+)	PM (-)	P value	PM (+)	PM (-)	P value
Sex			0.056			0.042*
Male	97 (28.4)	244 (71.6)		22 (48.9)	23 (51.1)	
Female	62 (29.4)	149 (70.6)		6 (24.0)	19 (76.0)	
Age at diagnosis			0.023*			0.075
< 60	99 (32.8)	203 (67.2)		20 (48.8)	21 (51.2)	
≥ 60	60 (24.0)	190 (76.0)		8 (27.6)	21 (72.4)	
Primary site			<0.001*			0.472
Right colon	63 (39.6)	96 (60.4)		11 (45.8)	13 (54.2)	
Left colon	70 (40.2)	104 (59.8)		17 (37.0)	29 (63.0)	
Rectum	26 (11.9)	193 (88.1)		–	–	
Histological type			<0.001*			0.367
AD	108 (23.3)	355 (76.7)		24 (37.5)	40 (62.5)	
MAD	38 (55.9)	30 (44.1)		2 (66.7)	1 (33.3)	
SRCC	13 (61.9)	8 (38.1)		2 (66.7)	1 (33.3)	
Differentiation			<0.001*			0.025*
Well/Moderate	82 (19.7)	335 (80.3)		21 (34.4)	40 (65.6)	
Poor/Undifferentiated	77 (57.0)	58 (43.0)		7 (77.8)	2 (22.2)	
T stage			<0.001*			0.472
T1-3	72 (21.7)	260 (78.3)		17 (37.0)	29 (63.0)	
T4	87 (39.5)	133 (60.5)		11 (45.8)	13 (54.2)	
N stage			0.014*			0.077
N0	31 (20.9)	117 (79.1)		12 (30.8)	27 (69.2)	
N1/N2	128 (31.7)	276 (68.3)		16 (51.6)	15 (48.4)	
Obstruction			<0.001*			0.024*
Negative	64 (16.7)	319 (83.3)		14 (30.4)	32 (69.6)	
Positive	95 (56.2)	74 (43.8)		14 (58.3)	10 (41.7)	
Perforation			0.041*			0.400
Negative	156 (28.5)	392 (71.5)		27 (39.1)	42 (60.9)	
Positive	3 (75.0)	1 (25.0)		1 (100)	0 (0)	
CEA			0.707			0.451
Nomal	74 (29.6)	176 (70.4)		13 (36.1)	23 (63.9)	
Elevated	85 (28.1)	217 (71.9)		14 (45.2)	17 (54.8)	
CA125			<0.001*			<0.001*
Nomal	81 (19.7)	330 (80.3)		7 (16.3)	36 (83.7)	
Elevated	78 (55.3)	63 (44.7)		21 (77.8)	6 (22.2)	
CA19-9			0.392			0.197
Nomal	102 (27.6)	267 (72.4)		19 (35.8)	34 (64.2)	
Elevated	57 (31.1)	126 (68.9)		7 (58.3)	5 (41.7)	
NLR			<0.001*			0.626
<2.5	55 (19.0)	235 (81.0)		15 (42.9)	20 (57.1)	
≥2.5	104 (39.7)	158 (60.3)		13 (37.1)	22 (62.9)	
PLR			<0.001*			0.329
<172.1	63 (20.7)	241 (79.3)		16 (45.7)	19 (54.3)	
≥172.1	96 (38.7)	152 (61.3)		12 (34.3)	23 (65.7)	
LMR			<0.001*			0.840
<2.6	89 (40.8)	129 (59.2)		10 (38.5)	16 (61.5)	
≥2.6	70 (21.0)	264 (79.0)		18 (40.9)	26 (59.1)	

PM, peritoneal metastasis; AD, adenocarcinoma; MAD, mucinous adenocarcinoma; SRCC, signet-ring cell carcinoma; CEA, carcinoembryonic antigen; CA125, carbohydrate antigen 125; CA19-9, carbohydrate antigen 19-9; NLR, neutrophil-lymphocyte ratio; PLR, platelet-lymphocyte ratio; LMR, lymphocyte-to-monocyte ratio.

P was calculated from univariate association of characteristics with occult PM status in colorectal cancer cohort; *P value < 0.05.

**Table 2:** Demographics of NRAS, KRAS, BRAF, PIK3CA and dMMR status.

Characteristics	Training cohort			External-validation cohort		
	PM (+)	PM (-)	P value	PM (+)	PM (-)	P value
NRAS			0.036*			–
Wild	61 (27.4)	162 (72.6)		5 (45.5)	6 (54.5)	
Mutation	3 (75.0)	1 (25.0)		–	–	
KRAS			0.546			>0.999
Wild	37 (29.8)	87 (70.2)		3 (50.0)	3 (50.0)	
Mutation	27 (26.2)	76 (73.8)		2 (40.0)	3 (60.0)	
BRAF			0.293			–
Wild	58 (27.4)	154 (72.6)		6 (42.9)	8 (57.1)	
Mutation	6 (40.0)	9 (60.0)		–	–	
PIK3CA			0.938			–
Wild	56 (28.3)	142 (71.7)		5 (55.6)	4 (44.4)	
Mutation	8 (27.6)	21 (72.4)		–	–	
dMMR			0.537			0.079
No	60 (27.8)	156 (72.2)		16 (40)	24 (60)	
Yes	4 (36.4)	7 (63.6)		5 (83.3)	1 (16.7)	

dMMR, mismatch-repair deficiency.

P was calculated from univariate association of characteristics with occult PM status in colorectal cancer cohort; *P value < 0.05.

### Construction and Validation of the Prediction Model

**Figure 1:**
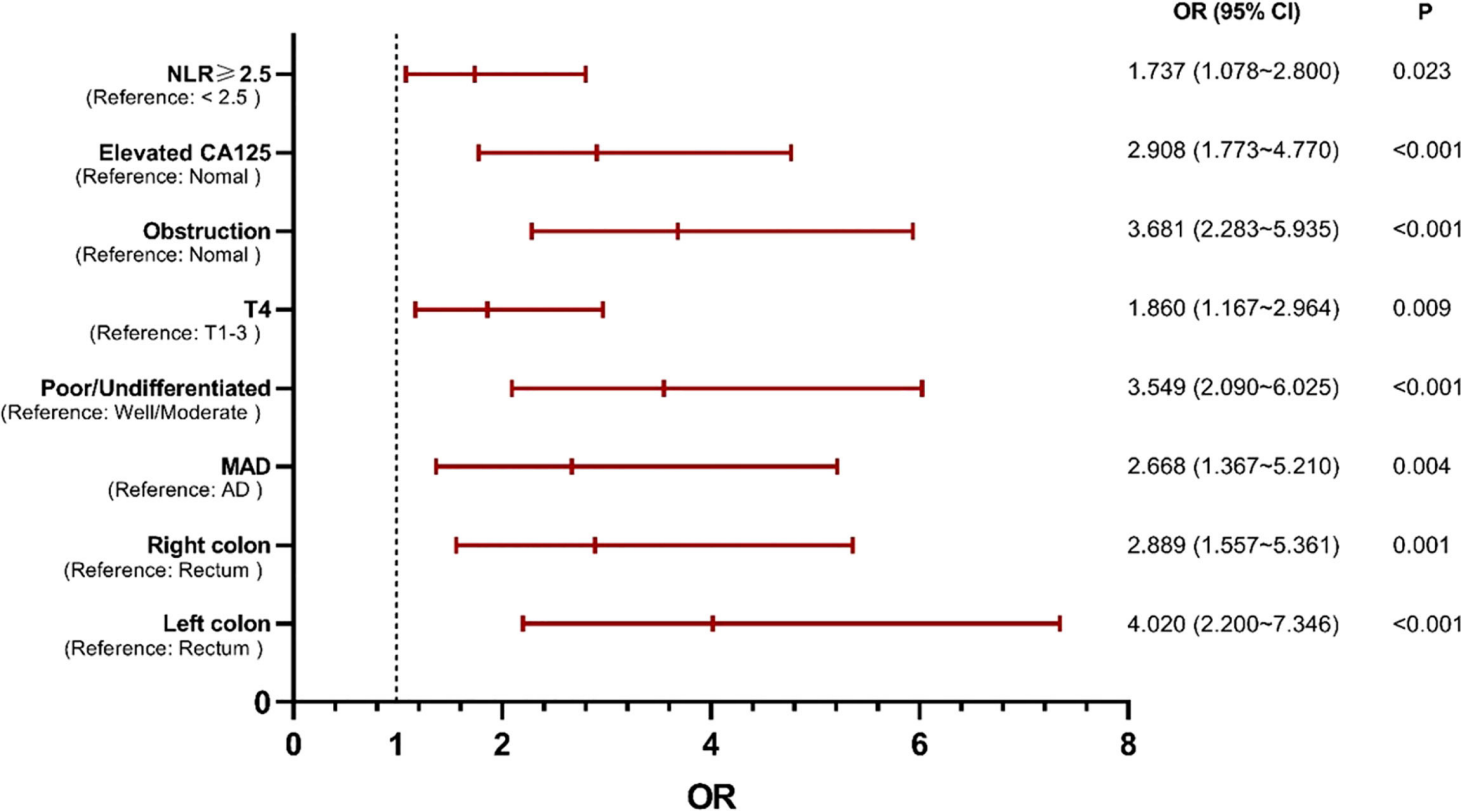
Independent predictors of occult PM identified by multivariate analysis. NLR, neutrophil-to-lymphocyte ratio; MAD, mucinous adenocarcinoma; AD, adenocarcinoma.

**Figure 2:**
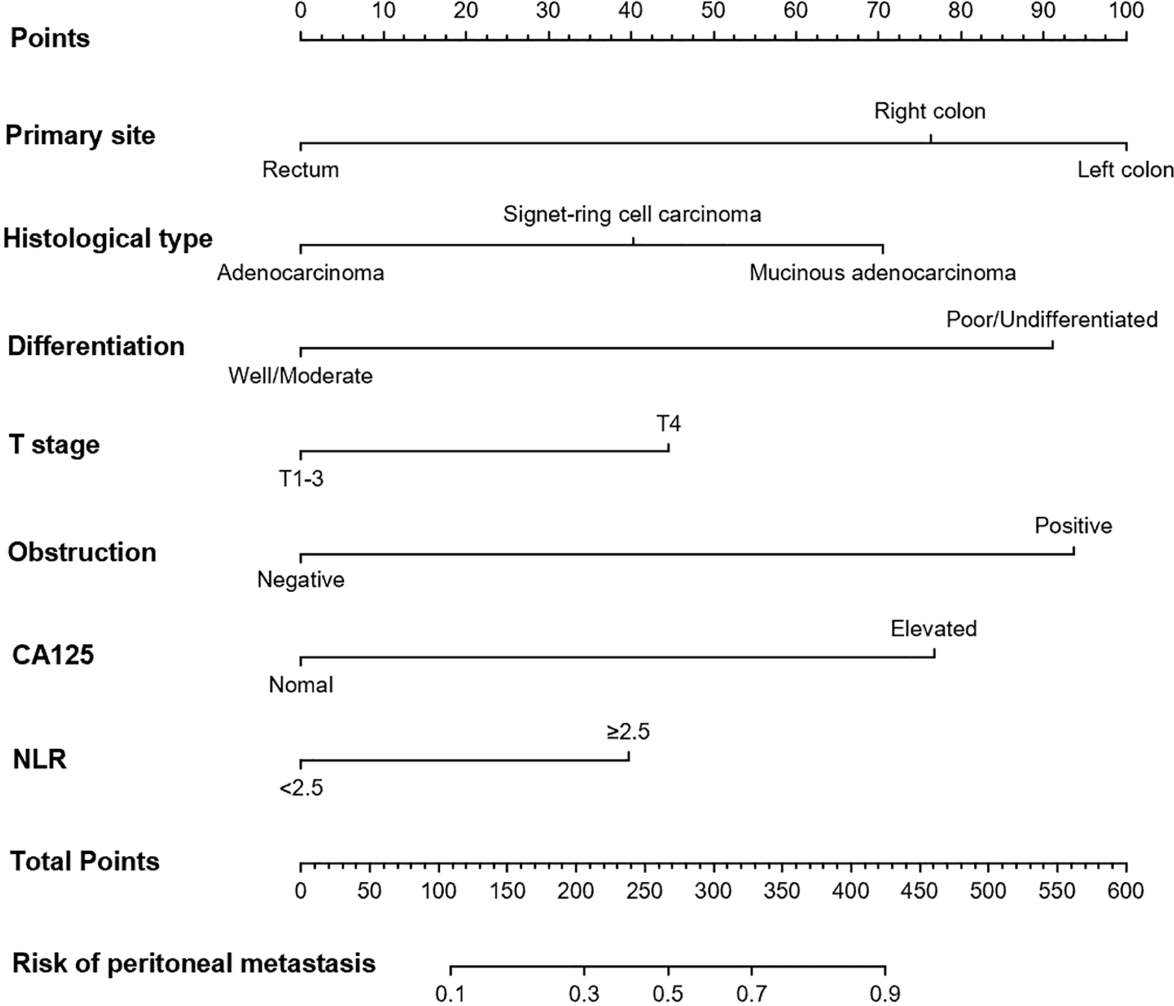
Nomogram for predicting the possibility of occult PM in CRC patients.

**Figure 3:**
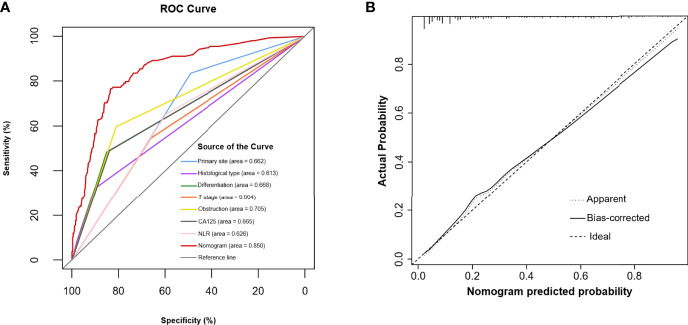
**(A)** The predictive accuracy of the model was assessed by a ROC curve. **(B)** Calibration curve of the prediction model.

**Figure 4:**
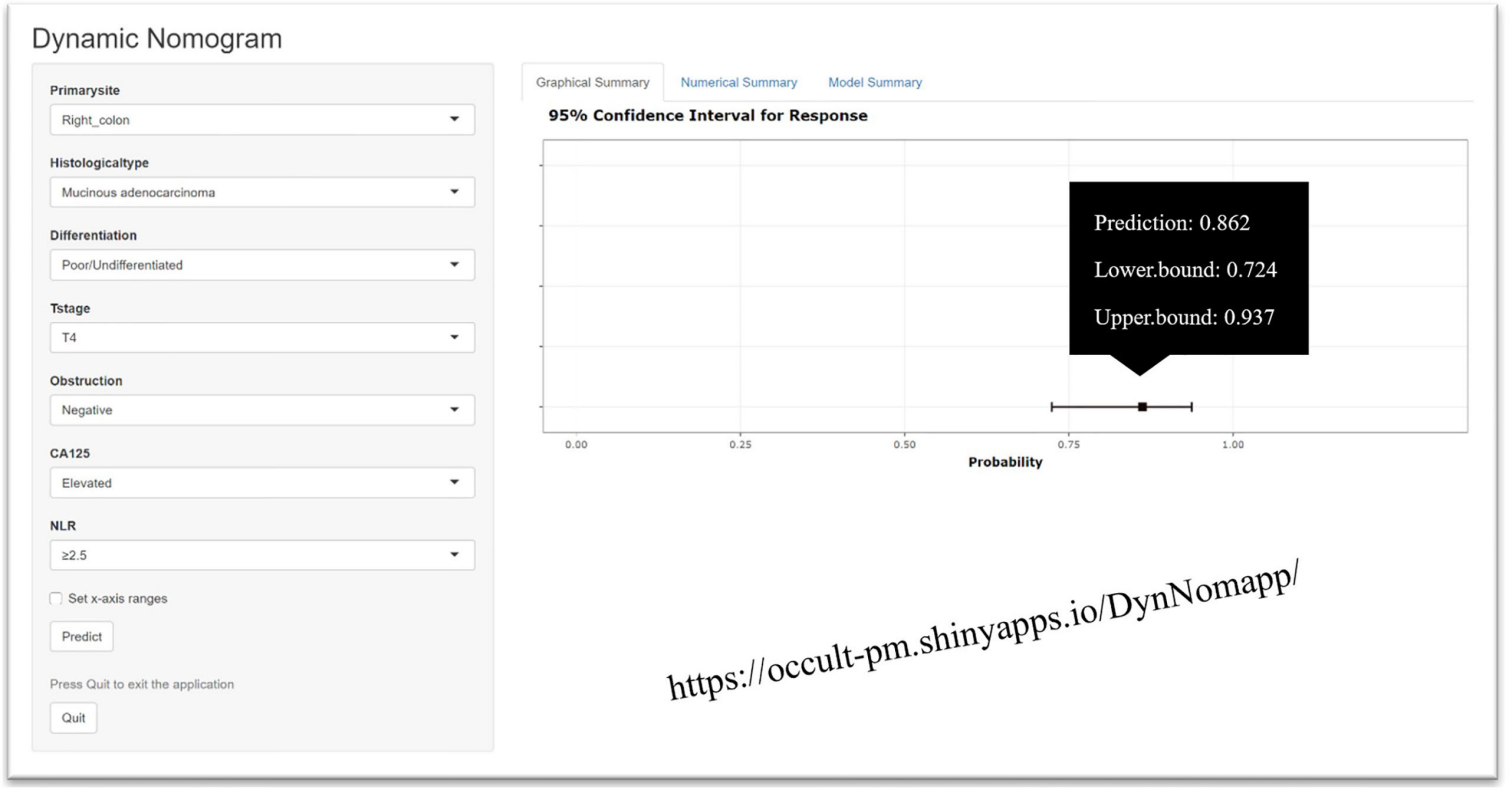
Webserver display of the dynamic online platform.

Multivariable analysis revealed that primary site, histological type, grade of differentiation, T stage, obstruction, serum CA125 and NLR were independent predictors of occult PM (**Figure 1**). Therefore, a predictive nomogram containing these variables was constructed (**Figure 2**). The Hosmer and Lemeshow test indicated a lack of significance (P = 0.841), demonstrating a good fit. In the training cohort, the AUC value of the prediction model was 0.850 (95% CI 0.815-0.885), and the ROC curve graphically showed that the model had better predictive performance than all univariate models alone (**Figure 3A**). The calibration curve of the model revealed good consistency between the prediction of occult PM and the actual situation observed (**Figure 3B**). A dynamic online platform (https://occult-pm.shinyapps.io/DynNomapp/) was developed to facilitate the usage of the proposed model (**Figure 4**). It can assist researchers and clinicians in more easily obtaining the risk probability of their patients by inputting their corresponding clinical variables, after which the web server will generate the output read in the form of figures and tables.

In the external validation cohort, the prediction model also yielded a high AUC of 0.794 (95% Cl, 0.690-0.899) (**Table 3**). This showed that the model could be applied to other independent patient populations.

**Table 3:** Performance evaluation of the prediction model.

Parameter	Training cohort	External-validation cohort
TP	122	24
TN	328	29
FN	37	4
FP	65	13
Sensitivity	0.767	0.857
Specificity	0.835	0.690
AUC	0.850 (0.815-0.885)	0.794 (0.690-0.899)
Risk cutoff, %	30^*^	–

TP, true positive; TN, true negative; FN, false negative; FP, false positive; AUC, area under curve.

*The cutoff value for probability threshold was set according to the maximum Youden index.

### Clinical Utility

**Figure 5:**
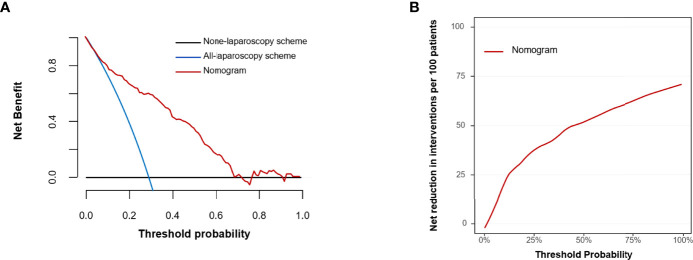
Decision curve analysis for the prediction model. **(A)** Net benefit. **(B)** Net reduction.

Based on a range of threshold probabilities, DCA was used to evaluate the clinical application of the prediction model. This analysis indicated that when the threshold probability was in the range between 0.10 and 0.72, using the model to predict occult PM would provide more benefits than using either the “treat all with laparoscopy” or “treat none with laparoscopy” plans (**Figure 5A**). The cutoff value for the probability threshold was set at 30%, according to the maximum Youden index (**Table 3**). DCA confirmed that the prediction model could improve risk stratification among patients with negative findings on peritoneal CT imaging by applying diagnostic laparoscopy to all or none of the CRC patients. For example, at the 30% risk cutoff, the prediction model’s net benefit was 60% (**Figure 5A**). We can state that if exploratory laparoscopy was performed when the patients’ risk threshold was >30%, compared to “treat none with laparoscopy”, the net benefit was equivalent to a net 60 true positive results per 100 patients without an increase in the number of false-positive results. Moreover, at a probability threshold of 30%, the net reduction in intervention was approximately 40 per 100 patients (**Figure 5B**). In other words, at this probability threshold, performing exploratory laparoscopy based on the model is equivalent to a strategy in which 40% of exploration could have been prevented without missing any occult PM cases.

## Discussion

The constructed model showed promising results with great predictive ability and clinical utility, improving exploratory laparoscopy risk stratification among CRC patients with negative findings on peritoneal CT imaging. In the training cohort, by choosing a risk cutoff of 30%, a total of 40% of exploratory laparoscopies could have been prevented while still identifying 76.7% of clinically occult PM cases.

Although some CT-based prediction models, including deep learning algorithms and spectral photon-counting imaging, have been established to diagnose PM, they still showed false-negative outcomes in small nodules and the proportion of false negatives increased with decreasing lesion size (21, 28). Furthermore, the tedious steps of obtaining CT sequence images and importing them into algorithm would limit the clinical application of these models. We believe that the early detection of PM is crucial, especially for occult micrometastatic lesions, because these nodules are not rich in blood supply and are usually missed on routine imaging. Importantly, exploratory laparoscopy is still the gold standard to confirm PM. Therefore, on the one hand, occult PM status could be identified based on our easy-to-use and individualized model, and optimal candidates for exploratory laparoscopy could be determined. On the other hand, *via* exploratory laparoscopy, physicians could evaluate the feasibility of CRS depending on the PCI and CCR (13, 22).

The “seed and soil” theory is a highly recognized theoretical mechanism of PM, and it suggests that PM initiation depends on the synergy of the tumor cells (seed) and the peritoneal microenvironment (soil) (29). A recent study on hallmarks of cancer further recognized that the tumor microenvironment plays an integral role in tumorigenesis and malignant progression (30). Our research team has been engaged in studying the tumor microenvironment as a factor associated with colorectal PM. We found that enhancing cancer-associated fibroblast fatty acid catabolism within a metabolically challenging tumor microenvironment drives colon cancer PM (31). Inflammation should also be recognized as a part of the microenvironment that could potentiate malignancy. The whole process of PM consists of a long series of sequential, interrelated steps. Exfoliated tumor cells in the peritoneal cavity need to maintain their metastatic potential; they need to prevent programmed cell death, and need motility to reach the peritoneal surfaces with the capacity to subsequently attach to these surfaces (32–34). Finally, the capacity to invade the peritoneum and survive and proliferate in the new environment is required (35, 36). Interestingly, peritoneal infections could enhance the migration, invasion, and even proliferation capacities of tumor cells *in vitro* (37). Moreover, experimental work has demonstrated the intimate connection between the molecules driving these steps and the inflammatory cytokines released due to infection (38–40). Especially in the early stages of colorectal PM, a major pattern of chronic inflammation represented by a tumor microenvironment rich in TAM2 with upregulation of IL-6 and rewiring of signaling pathways linked to plasticity, stemness and metabolism has been observed (41). Thus, inflammation-based factors such as obstruction and NLR may suggest early-stage or occult PM.

It is known that the major pathways of CRC cell dissemination are through direct transport, the lymphatic system and the circulatory system (42). N stage is traditionally believed to be the major milestone of tumor progression and is relevant to diverse metastatic sites. Interestingly, T4 neoplasms were significantly associated with PM, but the N stage was not an independent predictor for PM in this study. They are two variables that may have been confounded before. Advanced neoplasms present with rapid cell proliferation, increasing the interstitial fluid pressure in most solid tumors. Then, high pressure in the tumor increased the number of spontaneously shed tumor cells. This may lead to more tumor cells shedding into the lymphatics present in the tumor that can serve as a conduit for the tumor cells to metastasize to lymph nodes (43).

Several studies have reported that tumors carrying BRAF mutations exhibit a higher frequency of PM than tumors with a wild-type gene (2, 44). However, BRAF mutation was not significantly associated with occult PM in this study. The conflicting phenomenon may be that previous studies were designed to compare PM and non-PM, while our study was mainly based on comparing occult PM and non-PM. BRAF-mutant pmCRC was proven to exhibit a distinct pattern of metastatic spread (45, 46). Unlike an early-stage disease, these tumors are more likely to present with peritoneum-extensive metastasis, a pattern of metastatic spread that may contribute to the poor prognosis and survival of BRAF-mutant pmCRC patients. Only early-stage PM patients were enrolled in the cohort, and BRAF mutation was therefore noninformative in our model.

This study has some limitations. First, as with other retrospective studies, potential biases including case selection and model performance analysis, were inevitable. Second, although sequencing technology has become less expensive and tumor genotyping has become standard practice for pmCRC, the sample size of genes was relatively small, especially for mutation status, which may have limited its statistical power. Third, some studies found that the PCI score were underestimated for laparoscopy compared to laparotomy (13, 47). Even though it may underestimate PCI, exploratory laparoscopy accurately predicts the possibility of CCR. Finally, the threshold for exploratory laparoscopy should be determined after a physician and patient both weigh the harm of potentially unnecessary exploration and the benefit of confirming occult PM. Therefore, there is not a single risk threshold that can be used to determine who needs to undergo exploratory laparoscopy but rather a series of risk thresholds.

## Conclusions

Our individualized risk model can be used to reduce the number of unnecessary exploratory laparoscopies in patients who are unlikely to harbor clinically occult PM while capturing most of the patients with clinically occult PM. These results warrant further validation in prospective studies.

## Data Availability Statement

The dataset used during the study is available from the corresponding author on a reasonable request.

## Ethics Statement

This study was reviewed and approved by The Ethics Committees of The Sixth Affiliated Hospital of Sun Yat-sen University, No. 2020ZSLYEC-109. The requirement to obtain consent from patients was waived due to the retrospective nature of the study.

## Author Contributions

HW (9^th^ author) designed the study. YZ, XQ, VL, HW (10^th^ author) and SH contributed to the manuscript writing and revision. YL, ZW and XZ collected and summarized data. YZ and RL performed the statistical analysis and data interpretation. All authors read and approved the final draft.

## Funding

This study was sponsored by the National Natural Science Foundation of China (Grant No. 82103084) and the Sun Yat-sen University Clinical Research 5010 Program (Grant No. 2019021). The funding body had no role in the design of the study, the collection, analysis, and interpretation of the data or writing the manuscript.

## Conflict of Interest

The authors declare that the research was conducted in the absence of any commercial or financial relationships that could be construed as a potential conflict of interest.

## Publisher’s Note

All claims expressed in this article are solely those of the authors and do not necessarily represent those of their affiliated organizations, or those of the publisher, the editors and the reviewers. Any product that may be evaluated in this article, or claim that may be made by its manufacturer, is not guaranteed or endorsed by the publisher.

## Abbreviations

PM, peritoneal metastasis; CT, computed tomography; CRC, colorectal cancer; C-index, concordance index; ISRCTN, International Standard Randomised Controlled Trials Number; CRS, cytoreductive surgery; PCI, peritoneal cancer index; CCR, completeness of cytoreduction; CEA, carcinoembryonic antigen; CA125, carbohydrate antigen 125; CA19-9, carbohydrate antigen 19-9; dMMR, mismatch repair-deficient; NLR, neutrophil-to-lymphocyte ratio; PLR, platelet-to-lymphocyte ratio; LMR, lymphocyte-to-monocyte ratio; ROC, receiver operating characteristic; AUC, area under the ROC curve; DCA, decision curve analysis.

## References

[1] World Health Organization. International Agency for Research on Cancer. GLOBOCAN 2020: Estimated Cancer Incidence, Mortality and Prevalence Worldwide in 2020 . Available at: https://gco.iarc.fr/today.

[2] Franko J Shi Q Meyers JP Maughan TS Adams RA Seymour MT. Prognosis of Patients With Peritoneal Metastatic Colorectal Cancer Given Systemic Therapy: An Analysis of Individual Patient Data From Prospective Randomised Trials From the Analysis and Research in Cancers of the Digestive System (ARCAD) Database. Lancet Oncol (2016) 17:1709–19. doi: 10.1016/S1470-2045(16)30500-9 27743922

[3] Franko J Shi Q Goldman CD Pockaj BA Nelson GD Goldberg RM. Treatment of Colorectal Peritoneal Carcinomatosis With Systemic Chemotherapy: A Pooled Analysis of North Central Cancer Treatment Group Phase III Trials N9741 and N9841. JCO (2012) 30:263–7. doi: 10.1200/JCO.2011.37.1039 PMC3269953 22162570

[4] Klaver YLB Simkens LHJ Lemmens VEPP Koopman M Teerenstra S Bleichrodt RP. Outcomes of Colorectal Cancer Patients With Peritoneal Carcinomatosis Treated With Chemotherapy With and Without Targeted Therapy. Eur J Surg Oncol (2012) 38:617–23. doi: 10.1016/j.ejso.2012.03.008 22572106

[5] Elias D Glehen O Pocard M Quenet F Goéré D Arvieux C. A Comparative Study of Complete Cytoreductive Surgery Plus Intraperitoneal Chemotherapy to Treat Peritoneal Dissemination From Colon, Rectum, Small Bowel, and Nonpseudomyxoma Appendix. Ann Surg (2010) 251:896–901. doi: 10.1097/SLA.0b013e3181d9765d 20395843

[6] Glehen O Kwiatkowski F Sugarbaker PH Elias D Levine EA De Simone M. Cytoreductive Surgery Combined With Perioperative Intraperitoneal Chemotherapy for the Management of Peritoneal Carcinomatosis From Colorectal Cancer: A Multi-Institutional Study. JCO (2004) 22:3284–92. doi: 10.1200/JCO.2004.10.012 15310771

[7] Elias D Honoré C Dumont F Ducreux M Boige V Malka D. Results of Systematic Second-Look Surgery Plus HIPEC in Asymptomatic Patients Presenting a High Risk of Developing Colorectal Peritoneal Carcinomatosis. Ann Surg (2011) 254:289–93. doi: 10.1097/SLA.0b013e31822638f6 21709543

[8] Verwaal VJ Zoetmulder FAN. Follow-Up of Patients Treated by Cytoreduction and Chemotherapy for Peritoneal Carcinomatosis of Colorectal Origin. Eur J Surg Oncol (2004) 30:280–5. doi: 10.1016/j.ejso.2003.12.003 15028309

[9] Koh J-L Yan TD Glenn D Morris DL. Evaluation of Preoperative Computed Tomography in Estimating Peritoneal Cancer Index in Colorectal Peritoneal Carcinomatosis. Ann Surg Oncol (2009) 16:327–33. doi: 10.1245/s10434-008-0234-2 19050972

[10] Esquivel J Chua TC Stojadinovic A Melero JT Levine EA Gutman M. Accuracy and Clinical Relevance of Computed Tomography Scan Interpretation of Peritoneal Cancer Index in Colorectal Cancer Peritoneal Carcinomatosis: A Multi-Institutional Study. J Surg Oncol (2010) 102:565–70. doi: 10.1002/jso.21601 20976729

[11] Dromain C Leboulleux S Auperin A Goere D Malka D Lumbroso J. Staging of Peritoneal Carcinomatosis: Enhanced CT vs. PET/Ct. Abdom Imaging (2008) 33:87–93. doi: 10.1007/s00261-007-9211-7 17632751

[12] Bastiaenen VP Klaver CEL Kok NFM de Wilt JHW de Hingh IHJT Aalbers AGJ. Second and Third Look Laparoscopy in Pt4 Colon Cancer Patients for Early Detection of Peritoneal Metastases; the COLOPEC 2 Randomized Multicentre Trial. BMC Cancer (2019) 19:254. doi: 10.1186/s12885-019-5408-8 30898098 PMC6429794

[13] Najah H Malgras B Dohan A Gronnier C Eveno C Pocard M. The Role of Single-Incision Laparoscopic Peritoneal Exploration in the Management of Patients With Peritoneal Metastases. Surg Endosc (2020) 34:2040–9. doi: 10.1007/s00464-019-06984-8 31321535

[14] Goéré D. Incidence and Prognosis of Synchronous Colorectal Carcinomatosis: Evolution Since 1985? Future Oncol (2011) 7:1265–8. doi: 10.2217/fon.11.104 22044201

[15] Marmor RA Kelly KJ Lowy AM Baumgartner JM. Laparoscopy is Safe and Accurate to Evaluate Peritoneal Surface Metastasis Prior to Cytoreductive Surgery. Ann Surg Oncol (2016) 23:1461–7. doi: 10.1245/s10434-015-4958-5 26542584

[16] Tabrizian P Shrager B Jibara G Yang M-J Romanoff A Hiotis S. Cytoreductive Surgery and Hyperthermic Intraperitoneal Chemotherapy for Peritoneal Carcinomatosis: Outcomes From a Single Tertiary Institution. J Gastrointest Surg (2014) 18:1024–31. doi: 10.1007/s11605-014-2477-5 24577736

[17] Zarzavadjian le Bian A Genser L Denet C Ferretti C Laforest A Ferraz J-M. Safety and Feasibility of Repeat Laparoscopic Colorectal Resection: A Matched Case-Control Study. Surg Endosc (2020) 34:2120–6. doi: 10.1007/s00464-019-06995-5 31324972

[18] Cai X Duan L Wang Y Jiang W Liang X Yu H. Laparoscopic Hepatectomy by Curettage and Aspiration: A Report of 855 Cases. Surg Endosc (2016) 30:2904–13. doi: 10.1007/s00464-015-4576-0 26487222

[19] Cai XJ Yu H Liang X Wang YF Zheng XY Huang DY. Laparoscopic Hepatectomy by Curettage and Aspiration. Experiences of 62 Cases. Surg Endosc (2006) 20:1531–5. doi: 10.1007/s00464-005-0765-6 16865612

[20] Agha R Abdall-Razak A Crossley E Dowlut N Iosifidis C Mathew G. STROCSS 2019 Guideline: Strengthening the Reporting of Cohort Studies in Surgery. Int J Surg (2019) 72:156–65. doi: 10.1016/j.ijsu.2019.11.002 31704426

[21] Yuan Z Xu T Cai J Zhao Y Cao W Fichera A. Development and Validation of an Image-Based Deep Learning Algorithm for Detection of Synchronous Peritoneal Carcinomatosis in Colorectal Cancer. Ann Surg (2020) 275(4):e645–51. doi: 10.1097/SLA.0000000000004229 32694449

[22] Jacquet P Sugarbaker PH. Clinical Research Methodologies in Diagnosis and Staging of Patients With Peritoneal Carcinomatosis. Cancer Treat Res (1996) 82:359–74. doi: 10.1007/978-1-4613-1247-5_23 8849962

[23] Najah H Lo Dico R Eveno C Pocard M. Laparo-Endoscopic Single Site Surgery for Peritoneal Carcinomatosis Detection and Staging (With Video). J Visc Surg (2017) 154:133–4. doi: 10.1016/j.jviscsurg.2017.03.001 28395955

[24] Mandrekar JN. Receiver Operating Characteristic Curve in Diagnostic Test Assessment. J Thorac Oncol (2010) 5:1315–6. doi: 10.1097/JTO.0b013e3181ec173d 20736804

[25] On behalf of Topic Group ‘Evaluating diagnostic tests and prediction models’ of the STRATOS initiative Van Calster B McLernon DJ van Smeden M Wynants L Steyerberg EW. Calibration: The Achilles Heel of Predictive Analytics. BMC Med (2019) 17:230. doi: 10.1186/s12916-019-1466-7 31842878 PMC6912996

[26] Mehralivand S Shih JH Rais-Bahrami S Oto A Bednarova S Nix JW. A Magnetic Resonance Imaging-Based Prediction Model for Prostate Biopsy Risk Stratification. JAMA Oncol (2018) 4:678–85. doi: 10.1001/jamaoncol.2017.5667 PMC5885194 29470570

[27] Vickers AJ Elkin EB. Decision Curve Analysis: A Novel Method for Evaluating Prediction Models. Med Decis Making (2006) 26:565–74. doi: 10.1177/0272989X06295361 PMC2577036 17099194

[28] Thivolet A Si-Mohamed S Bonnot P-E Blanchet C Képénékian V Boussel L. Spectral Photon-Counting CT Imaging of Colorectal Peritoneal Metastases: Initial Experience in Rats. Sci Rep (2020) 10:13394. doi: 10.1038/s41598-020-70282-w 32770125 PMC7414131

[29] Fidler IJ. The Pathogenesis of Cancer Metastasis: The “Seed and Soil” Hypothesis Revisited. Nat Rev Cancer (2003) 3:453–8. doi: 10.1038/nrc1098 12778135

[30] Hanahan D. Hallmarks of Cancer: New Dimensions. Cancer Discovery (2022) 12:31–46. doi: 10.1158/2159-8290.CD-21-1059 35022204

[31] Peng S Chen D Cai J Yuan Z Huang B Li Y. Enhancing Cancer-Associated Fibroblast Fatty Acid Catabolism Within a Metabolically Challenging Tumor Microenvironment Drives Colon Cancer Peritoneal Metastasis. Mol Oncol (2021) 15:1391–411. doi: 10.1002/1878-0261.12917 PMC8096782 33528867

[32] Fermor B Umpleby HC Lever JV Symes MO Williamson RC. Proliferative and Metastatic Potential of Exfoliated Colorectal Cancer Cells. J Natl Cancer Inst (1986) 76:347–9. 3456069

[33] Umpleby HC Fermor B Symes MO Williamson RCN. Viability of Exfoliated Colorectal Carcinoma Cells. Br J Surg (2005) 71:659–63. doi: 10.1002/bjs.1800710902 6478151

[34] Symes MO Fermor B Umpleby HC Tribe CR Williamson RC. Cells Exfoliated From Colorectal Cancers can Proliferate in Immune Deprived Mice. Br J Cancer (1984) 50:423–5. doi: 10.1038/bjc.1984.193 PMC1976787 6466549

[35] de Cuba EMV Kwakman R van Egmond M Bosch LJW Bonjer HJ Meijer GA. Understanding Molecular Mechanisms in Peritoneal Dissemination of Colorectal Cancer: Future Possibilities for Personalised Treatment by Use of Biomarkers. Virchows Arch (2012) 461:231–43. doi: 10.1007/s00428-012-1287-y 22825001

[36] Klaver CEL Wasmann KATGM Verstegen M van der Bilt JDW Nagtegaal ID van Ramshorst B. Postoperative Abdominal Infections After Resection of T4 Colon Cancer Increase the Risk of Intra-Abdominal Recurrence. Eur J Surg Oncol (2018) 44:1880–8. doi: 10.1016/j.ejso.2018.09.016 30360990

[37] Salvans S Mayol X Alonso S Messeguer R Pascual M Mojal S. Postoperative Peritoneal Infection Enhances Migration and Invasion Capacities of Tumor Cells *In Vitro*: An Insight Into the Association Between Anastomotic Leak and Recurrence After Surgery for Colorectal Cancer. Ann Surg (2014) 260:939–44. doi: 10.1097/SLA.0000000000000958 25243554

[38] Coussens LM Werb Z. Inflammation and Cancer. Nature (2002) 420:860–7. doi: 10.1038/nature01322 PMC2803035 12490959

[39] Aggarwal BB Vijayalekshmi RV Sung B. Targeting Inflammatory Pathways for Prevention and Therapy of Cancer: Short-Term Friend, Long-Term Foe. Clin Cancer Res (2009) 15:425–30. doi: 10.1158/1078-0432.CCR-08-0149 19147746

[40] Wu Y Zhou BP. Inflammation: A Driving Force Speeds Cancer Metastasis. Cell Cycle (2009) 8:3267–73. doi: 10.4161/cc.8.20.9699 PMC3702728 19770594

[41] Barriuso J Nagaraju RT Belgamwar S Chakrabarty B Burghel GJ Schlecht H. Early Adaptation of Colorectal Cancer Cells to the Peritoneal Cavity Is Associated With Activation of “Stemness” Programs and Local Inflammation. Clin Cancer Res (2021) 27:1119–30. doi: 10.1158/1078-0432.CCR-20-3320 PMC7611320 33257424

[42] Ceelen WP Bracke ME. Peritoneal Minimal Residual Disease in Colorectal Cancer: Mechanisms, Prevention, and Treatment. Lancet Oncol (2009) 10:72–9. doi: 10.1016/S1470-2045(08)70335-8 19111247

[43] Hayashi K Jiang P Yamauchi K Yamamoto N Tsuchiya H Tomita K. Real-Time Imaging of Tumor-Cell Shedding and Trafficking in Lymphatic Channels. Cancer Res (2007) 67:8223–8. doi: 10.1158/0008-5472.CAN-07-1237 17804736

[44] Lipsyc M Yaeger R. Impact of Somatic Mutations on Patterns of Metastasis in Colorectal Cancer. J Gastrointest Oncol (2015) 6:645–9. doi: 10.3978/j.issn.2078-6891.2015.045 PMC4671855 26697197

[45] Tran B Kopetz S Tie J Gibbs P Jiang Z-Q Lieu CH. Impact of BRAF Mutation and Microsatellite Instability on the Pattern of Metastatic Spread and Prognosis in Metastatic Colorectal Cancer. Cancer (2011) 117:4623–32. doi: 10.1002/cncr.26086 PMC4257471 21456008

[46] Yokota T Ura T Shibata N Takahari D Shitara K Nomura M. BRAF Mutation is a Powerful Prognostic Factor in Advanced and Recurrent Colorectal Cancer. Br J Cancer (2011) 104:856–62. doi: 10.1038/bjc.2011.19 PMC3048210 21285991

[47] Passot G Dumont F Goéré D Arvieux C Rousset P Regimbeau J-M. Multicentre Study of Laparoscopic or Open Assessment of the Peritoneal Cancer Index (BIG-RENAPE). Br J Surg (2018) 105:663–7. doi: 10.1002/bjs.10723 29579322

